# An extremely dangerous case of acute massive upper gastrointestinal bleeding: a case report

**DOI:** 10.1186/s12876-022-02138-8

**Published:** 2022-02-15

**Authors:** Zhiqiang Yi, Cheng Chen, Biguang Tuo, Taolang Li, Xuemei Liu

**Affiliations:** 1 Department of Gastroenterology, Affiliated Hospital of Zunyi Medical University, Zunyi, 563003 Guizhou Province China; 2 Department of Thoracis Surgery, Affiliated Hospital of Zunyi Medical University, Zunyi, Guizhou Province China; 3 Department of Thyroid and Breast Surgery, Affiliated Hospital of Zunyi Medical University, Zunyi, Guizhou Province China

**Keywords:** Delayed but high-risk massive upper gastrointestinal bleeding, Fish bone, Mediastinal abscess, Left subclavian artery (LSA), Early and timely multidisciplinary collaboration

## Abstract

**Background:**

Upper gastrointestinal (GI) bleeding is a severe acute disease of gastroenterology department. Fish bone is the most common food-related foreign body. However, fish bone piercing the esophagus, causing the mediastinal abscess that corroded the left subclavian artery, resulting delayed but high-risk massive upper gastrointestinal bleeding is very rare.

**Case presentation:**

We report a 54-year-old man who was diagnosed with delayed but high-risk massive upper GI bleeding that was the result of a fish bone piercing the esophagus, causing a mediastinal abscess that corroded the left subclavian artery. He was saved effectively by early and timely multidisciplinary collaboration.

**Conclusion:**

A fish bone-caused mediastinal abscess that corrodes the left subclavian artery and induces delayed but high-risk massive upper GI bleeding is very rare. In addition to routine consideration of upper GI bleeding, medical history, endoscopy and CT are helpful for achieving a diagnosis. Importantly, early and timely multidisciplinary collaboration can effectively save critically ill patients.

## Background

Upper gastrointestinal (GI) bleeding is a severe acute disease associated with four main causes, namely, esophageal variceal bleeding, peptic ulcer (PU) bleeding, gastric cancer bleeding and acute erosive hemorrhagic gastritis, and prompt diagnosis and treatment are mandatory [1, 2]. In adults, a fish bone is the most common food-related foreign body [3, 4]. A fish bone can unfortunately pierce the left subclavian artery (LSA) and cause a pseudoaneurysm in rare cases, resulting in an LSA esophageal fistula [3, 5]. Arterioesophageal fistula are rare, but they can cause massive life-threatening bleeding [6, 7]. Here, we report an adult case of a mediastinal abscess corroding the LSA, resulting in delayed but high-risk massive upper GI bleeding.

## Case presentation

A 54-year-old man was admitted to the hospital with a chief complaint of hematemesis for 2 h. The color of hematemesis was bright red, and the volume was over 1000 ml. He also had symptoms of palpitations, fatigue and sweating. He had been on long-term glucocorticoids or nonsteroidal anti-inflammatory drugs (NSAIDs) for gout for 3 years before admission, had no previous history of liver disease or GI bleeding disease, and had a history of drinking.

**Fig. 1:**
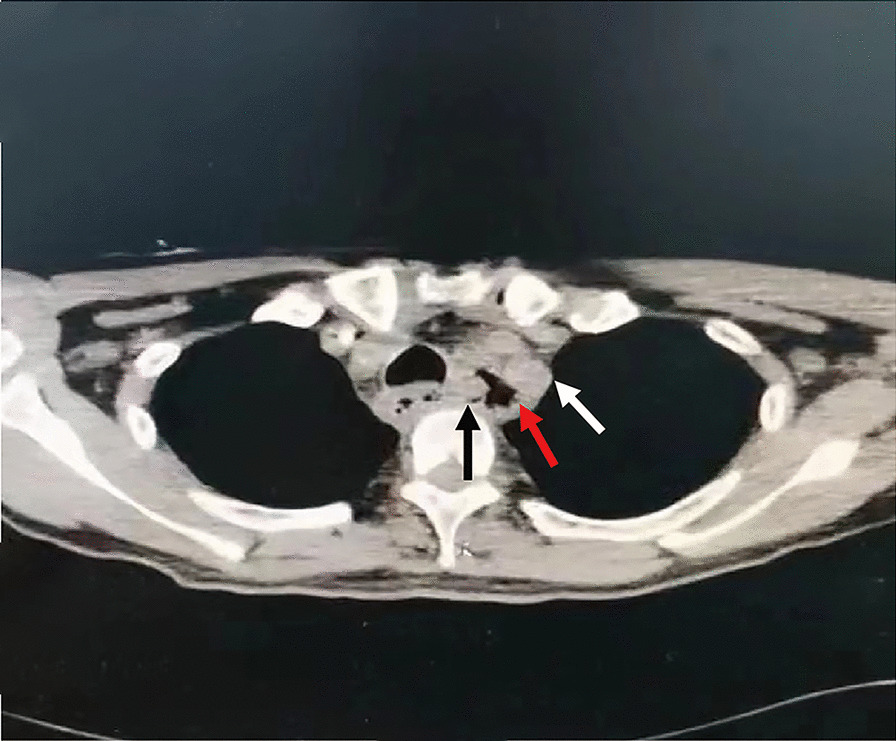
Image from CT scan. CT showed that the local soft tissue was thickened in the esophagus at the cervicothoracic junction, and a gaseous cavity was present on the left posterior wall of the esophagus (red arrow), approaching the left subclavian artery (white arrow). Esophageal breach was detected (black arrow)

**Fig. 2:**
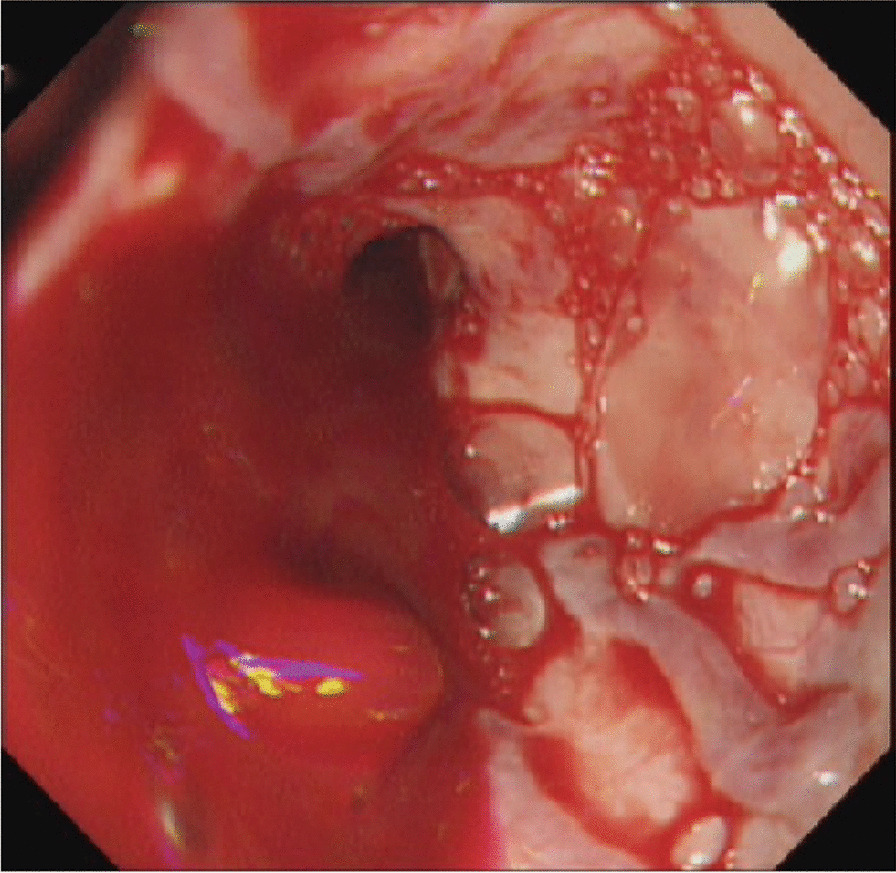
Image from the emergent endoscopy. Active and massive bleeding was detected from the esophageal wall by emergent endoscopy

On admission, the patient's vital signs included a temperature of 36.4 °C, blood pressure of 80/50 mmHg, and heart rate of 135 bpm. Laboratory data showed routine blood tests: hemoglobin 72 g/L (normal, 130–175 g/L), white blood cells 13.41 × 10^9^/l (normal, 4–10 * 10^9^/L), total platelets 60 × 10^9^ (normal, 100–300 * 10^9^/L); C-reactive protein 159.90 mg/l (normal 0–8 mg/L); blood gas analysis: oxygen partial pressure 79.3 mmHg, CO_2_ partial pressure 30.8 mmHg, pH value 7.281; and normal liver and renal function and coagulation tests. Therefore, GI hemorrhage was first considered, and esomeprazole was administered. Computed tomography (CT) on admission showed a visible breach on the left side of the esophageal wall, a low-density lesion was found between the esophageal wall and the LSA, and the demarcation from the esophageal wall and subclavian artery was unclear (Fig. 1). Because the patient had no history of foreign body ingestion, we initially considered esophageal diverticula with bleeding potential. However, the patient still presented with repeated hematemesis with a large volume of bright red blood; his vital signs could not be maintained, and he developed progressive unconsciousness 30 min after admission to the hospital. We applied a multidisciplinary approach; critical care doctors were included in the treatment, and a member of the Department of Anesthesiology performed an urgent consultation. Tracheal intubation-assisted respiration, aspiration prevention, and anti-shock therapy were applied, and 6 units of red blood cells were transfused. Additionally, urgent esophagogastroduodenoscopy was performed in the operating room, which indicated active and massive bleeding from the esophageal wall (Fig. 2). However, due to massive bleeding, this patient suffered from two cardiac arrests, so a detailed endoscopic examination could not be performed.

**Fig. 3:**
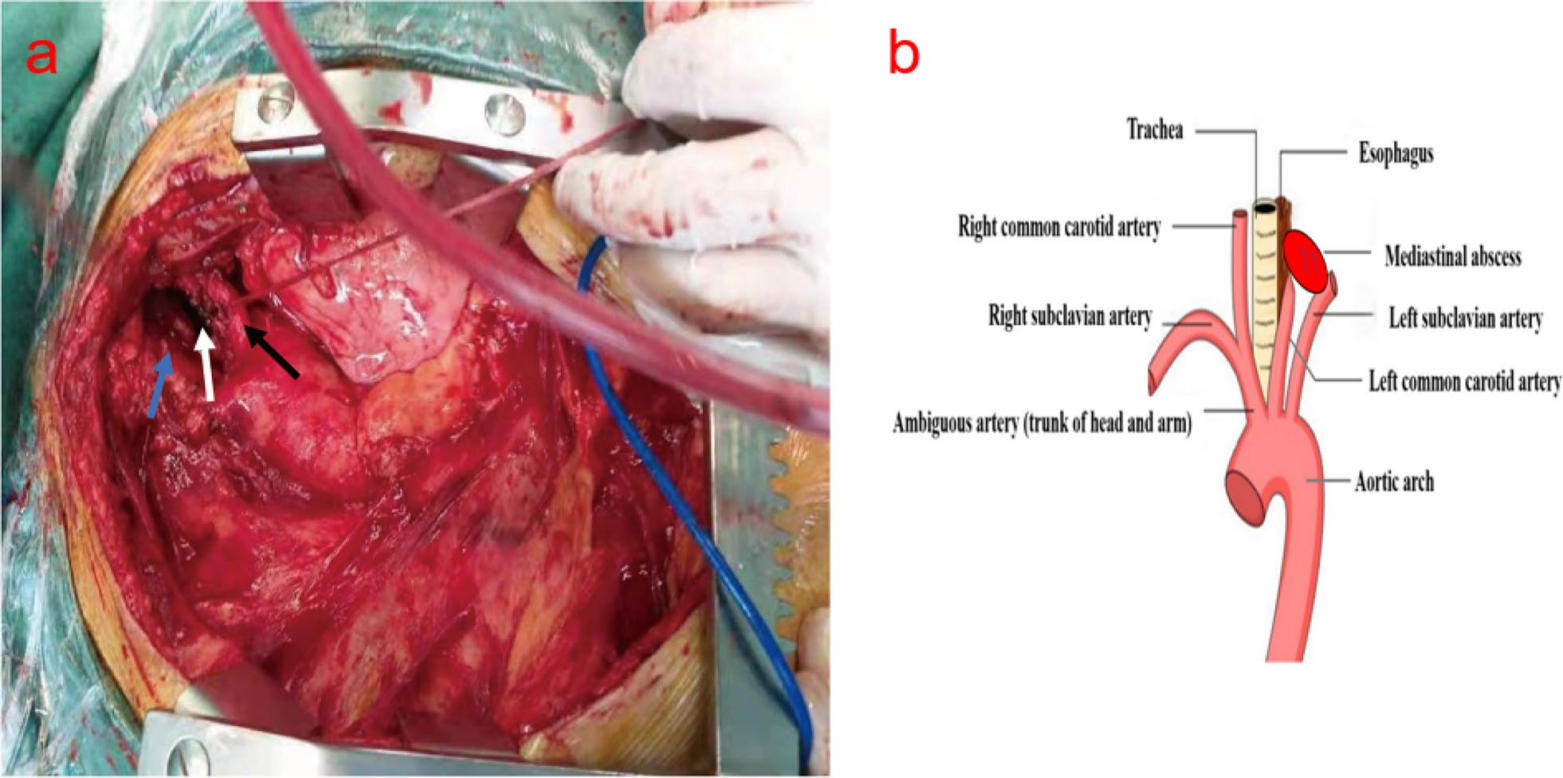
Image from the surgical operation. **a** The abscess cavity (white arrow) was located between the left subclavian artery (black arrow) and the left common carotid artery (blue arrow). **b** Schematic diagram of surgical anatomy

Urgent thoracotomy through the median sternal incision was performed immediately, and an abscess cavity 4 cm in size was detected between the LSA and the left common carotid artery. A stench of putrefaction accompanied by a large gush of blood was present when the abscess cavity was opened. The active bleeding point was found in the posterior lateral wall of the LSA with a 3 mm rupture. During the repair of the LSA and clean-up of the abscess cavity, an esophageal fistula was detected and repaired on the other side of the abscess cavity. However, no foreign body was observed (Fig. 3a, b). With prompt and effective treatment, the patient recovered. Revisiting family history indicated that chest pain and discomfort occurred in this patient after eating fish 4 days prior.

## Discussion and conclusions

There are generally four main causes of upper GI bleeding, namely, esophageal variceal bleeding, PU-caused bleeding, gastric cancer-caused bleeding and acute erosive hemorrhagic gastritis [1, 2]. In addition, there are also reports of NSAIDs causing esophageal mucosal damage. These drugs may cause mucosal damage by reducing the cytoprotective effect of prostaglandins on the mucosa or aggravating reflux esophagitis [8, 9]. The clinical manifestations of these patients are usually retrosternal pain, sore throat and dysphagia [9]. Esophageal bleeding caused by NSAIDs is a rare nonfatal bleeding, mostly secondary to superficial esophageal ulcer oozing, and some bleeding can even be discovered during endoscopy [10, 11]. However, in the present case, the patient's condition progressed so rapidly and dangerously that the challenge was to determine the cause of the bleeding and how to treat it. Based on medical history, physiological signs, examination and treatment, this case of delayed but high-risk massive upper GI bleeding was the result of a fish bone piercing the esophagus, causing the mediastinal abscess that corroded the LSA. Fistulas between the subclavian artery and esophagus are rare but can rapidly become life-threatening [12, 13]. Clinically, among arterial-esophageal fistulas caused by esophageal foreign bodies, the proportion of LSA esophageal fistulas is only 2% [3]. Most mediastinal abscesses are related to infections of deep sternal wounds, esophageal perforations, or descending necrotizing mediastinitis [14, 15]. Mediastinal abscess directly caused by taking glucocorticoids has not been reported. It may be a contributing factor to the formation of abscesses after mediastinal infection. Glucocorticoids have many complex quantitative and qualitative immunosuppressive effects, which can induce cellular immune deficiency and may increase the susceptibility of the host to various viruses, bacteria, fungi and parasites [16]. The patient’s risk of infection increases with increasing treatment dose and treatment time. In patients exposed to low doses, even if the cumulative dose is high, the risk of infection is often low. In addition, the host's underlying disease status also determines the changes in the risk of infection in clinical practice [17]. Therefore, in this case, we believe that the patient's long-term use of glucocorticoids is not an independent risk factor for mediastinal abscess.

Generally, fish bones or sharp foreign bodies can directly pierce the large arteries in the mediastinum, causing fatal upper GI bleeding. For example, some case reports have reported sharp foreign bodies such as fish bones or chicken bones piercing the esophagus and mediastinal artery [3, 18, 19]. Because the symptoms are initially not serious, they are often ignored by patients and their families. Once the foreign body leaves the artery, the patient will die suddenly due to acute upper GI bleeding [3, 18]. If the patient seeks medical attention in time and the foreign body does not break away from the artery, the patient can be successfully cured by surgery [19]. In contrast, in this rare case, the mediastinal abscess that resulted from puncture of the esophagus by the fishbone corroded the LSA, causing delayed but high-risk arterial hemorrhage.

This case suggests that in addition to routine consideration of upper GI bleeding, medical history, endoscopy and CT are helpful for achieving a diagnosis. Importantly, early and timely multidisciplinary collaboration can effectively save critically ill patients. Emergency thoracotomy remains a life-saving treatment.

## Data Availability

All information about the patient came from the Affiliated Hospital of Zunyi Medical University. The data used and analyzed during the current study are included in this article.

## Abbreviations

GI: Gastrointestinal
NSAIDs: Nonsteroidal anti-inflammatory drugs
LSA: Left subclavian artery
CT: Computed tomography
PU: Peptic ulcer
